# A Practical Treatise on Fractures and Dislocations

**Published:** 1884-12

**Authors:** 


**A Practical Treatise on Fractures and Dislocations.** By Frank Hastings Hamilton, M. D., LL. D., late Professor of Surgery in Bellevue Hospital Medical College, etc. Seventh American edition, revised and improved. Illustrated with 379 wood-cuts. Philadelphia: H. C. Lea's Son & Co. 1884.

Dr. Hamilton's classical work needs no introduction, as it has held its own in professional favor throughout the last twenty years. It is sufficient to say that this last edition gives evidence of having been revised with elaborate care by its distinguished author. Beside the numerous additions drawn from his more recent personal experience, he has brought together a great number of the more lately recorded facts and observations relating to his subject. This edition is thereby considerably enlarged and proportionately increased in value. This admirable edition of the work ensures its continued recognition as the most complete and practical treatise on the subject. With regard to the mechanical execution of the work, we could not suggest an improvement. The illustrations are admirable and abundant.

